# New Bills of Mortality for London

**Published:** 1840-10

**Authors:** 


					1840.] 58?
PART FOURTH.
JWrtitcal 3ntelUgewe*
TABLE OF MORTALITY FOR LONDON,
For the Second Quarter of 1840:
Showing the number of Deaths from all causes registered in thirteen weeks, from the
4th April to the 27th June, 1840.
Causes of Death.
April.
Week ending
4th 11th 18th 25th
May.
Week ending
2d 9th 16th 23d 30th
June.
Week ending
6th 13th 20th 27th
Total.
Class r.
Smallpox
Measles
Scarlatina
Hooping-cough
Croup
Thrush
Diarrhoea
Dysentery
Cholera
Influenza
Typhus
Erysipelas
Syphilis
Hydrophobia
Total Epidemic, &c.
Class ii.
Cephalitis
Hydrocephalus
Apoplexy
Paralysis
Convulsions
Epilepsy
Insanity
Delirium tremens
Diseases of the brain, &c..
Total Dis. of Brain, &c
Class nr.
Quinsey
Bronchitis
Pleurisy
Pneumonia
Hydrothorax
Asthma
Consumption
Diseases of the lungs, &c.
Total Dis. of Lungs, &c.
Class iv.
Pericarditis
Aneurism
Diseases of the heart, &c...
Total Dis. of Heart, &c.
169
19
147
157
167
165
137
157
274
238 220
217
231 224
VOL. X. NO.' XX. *19
590 Medical Intelligence. [Oct.
Causes of Death.
April.
Week ending
4th |llth|18th|25th
Class v.
Teething
Gastritis, enteritis ....
Peritonitis
Tabes mesenterica ...
Ascites
Ulceration
Hernia
Colic or ileus
Diseases of the stomach,&c.
Hepatitis
Jaundice
Disease of the liver, &c. ..
Total Dis.of Stomach, &c.
Class vi.
Nephritis
Diabetes
Stone
Stricture
Diseases of the kidneys,&c.
Total Dis. of Kidneys, &c.
Class vir.
Childbed
Ovarian dropsy
Diseases of uterus, &c..
Total Dis. of Uterus, &c.
Class vtii.
Rheumatism
Diseases of joints, &c. .
Total Dis. of Joints, &c.
Classix.
Ulcer
Fistula
Diseases of skin, &c. .
Total Dis. of Skin, &c.
Class x.
Inflammation
Hemorrhage
Dropsy
Abscess
Mortification
Scrofula
Carcinoma
Tumour
Gout
Atrophy
Debility
Malformations
Sudden death
Total Dis. of Uncertain Seat.
Class xi.
Old age or natural decay ..
Class xn.
Intemperance
Privation
Violent deaths
Total by Violence, &c.
Class xm.
Causes not specified ....
Total Deaths from all causes
78 85
48
14
16
3]
6
?1
0
2 01
145 87
97
31
32
May.
Week ending
2d , 9th ,16th 123d |30th
57
106
67
68
23
66
37
12
27,
120
62
62
June.
Week ending
6th |13th|20th|28th
102 108:
49
9761 851 885 888| 853| 830| 853( 756| 795| 785 819 771 824 10886
16
104
56
10
'95 110
21
Total.
1840.] The Respirator. 591
NUMBER OF DEATHS IN THE DIFFERENT DISTRICTS.
Districts.
West Districts
North Districts
Central Districts
East Districts
South Districts
Total (Males 5716, Females 5170)
Estimated Population
in 1840.
308,921
414,458
369,722
411,634
450,265
1,955,000
Deaths during
the Quarter.
1738
2029
2112
2256
2751
10,886

				

